# Kiwifruit Bacterial Canker Susceptibility of 86 Accessions and Their Physiological Response to Psa Inoculation

**DOI:** 10.3390/plants15162494

**Published:** 2026-08-18

**Authors:** Mengjie Chen, Jiale Tang, Sha Mo, Rencai Wang, Feixiong Luo

**Affiliations:** 1 College of Horticulture, Hunan Agricultural University, Changsha 410128, China; 2 Hunan Academy of Agricultural Sciences, Changsha 410125, China; 3 Yuelushan Laboratory, Changsha 410128, China

**Keywords:** *Actinidia*, bacterial canker, detached shoot inoculation, germplasm evaluation, defense enzyme, ROS homeostasis, disease-resistant breeding

## Abstract

Kiwifruit bacterial canker, caused by *Pseudomonas syringae* pv. *actinidiae* (Psa), severely restricts the sustainable development of the kiwifruit industry. Screening stable resistant germplasm and establishing efficient disease resistance evaluation methods are core prerequisites for breeding resistant cultivars. In this study, 86 *Actinidia* accessions were systematically assessed for Psa susceptibility over three consecutive years using the reported detached shoot inoculation assay. Seven representative accessions with contrasting resistance phenotypes, namely ‘Cuiyu’, ‘Chuhong’, ‘Jinmei’, ‘Hongyang’, ‘Cuixiang’, ‘Avfs08’, and ‘G3’, were selected to measure the activities of four defense-related enzymes post Psa inoculation to dissect the physiological mechanisms driving divergent Psa resistance in kiwifruit. Lesion lengths across years exhibited a significant positive correlation, demonstrating that this inoculation method delivers repeatable, genetically stable phenotypic data with limited environmental interference. Two accessions belonging to *A. valvata* and *A. eriantha* exhibited stable high resistance via synergistic biochemical defenses. By contrast, the widely grown cultivar ‘Hongyang’ was highly susceptible, while moderately resistant materials such as ‘Cuiyu’ and ‘Yannong 3’ were discovered within the inherently susceptible species *A. chinensis*. Highly resistant accessions rapidly induced coordinated increases in SOD and PAL activity at 24 h post inoculation to maintain ROS homeostasis and lignin biosynthesis, whereas susceptible accessions displayed chaotic, ineffective enzymatic stress responses. Temporal synergy of PAL and POD may act as the key defensive regulatory mode. This study uncovered substantial interspecific variation in Psa resistance across *Actinidia* germplasm, identified elite donors with stable resistance, and elucidated the physiological mechanisms of kiwifruit resistance to Psa. These findings provided a theoretical foundation and valuable germplasm for subsequent resistance gene mining and disease resistance breeding.

## 1. Introduction

Kiwifruit are rich in ascorbic acid and diverse nutraceutical bioactive metabolites, possessing high commercial value and health-promoting properties for humans, and are cultivated worldwide as economically important fruit crops. However, continuous expansion of planting area and genetic homogenization of a few cultivars have elevated phytosanitary risks, which have become a major bottleneck that restricts the quality and sustainable development of the kiwifruit industry. Several diseases limit kiwifruit production across the world, among which kiwifruit vine decline syndrome (KVDS) is a complex multifactorial disorder induced by soil-borne oomycete pathogens and waterlogging-induced hypoxia [1,2]. Of all these diseases, bacterial canker caused by *Pseudomonas syringae* pv. *actinidiae* (Psa) ranks as the most destructive and prevalent disease, inflicting severe economic losses on kiwifruit-growing regions worldwide [3]. Since its pandemic outbreak in Italy in 2008 [4], Psa has spread rapidly across major kiwifruit-producing regions worldwide [5,6,7]. Under favorable climatic conditions, the pathogen induces branch cankers, whole-plant death, and even total orchard collapse within a short infection period, posing severe threats to the global kiwifruit industry [3].

Psa infection causes tissue- and organ-specific symptoms in kiwifruit. Stem cankers are the most typical lesion phenotype: at the early infection stage, bark tissues show water-soaked rotting accompanied by milky-white bacterial exudates. These exudates slowly turn reddish-brown after oxidation and leave dark brown residues upon drying. Severe infection leads to longitudinal bark splitting and reddish xylem necrosis [8,9,10]. Leaf symptoms start as polygonal necrotic brown spots, usually 1 mm ~ 3 mm in diameter, surrounded by chlorotic halos that are usually 3 mm ~ 5 mm wide, followed by leaf curling and marginal scorch. Psa-induced flower blight further results in floral necrosis, reduced fruit set, fruit malformation, and embryo abortion [11,12]. Biovar 3 (Psa3) is the dominant pathotype of global Psa populations at present, prevailing in major kiwifruit growing areas and causing massive economic losses in commercial kiwifruit production [5,13,14].

The genus *Actinidia* harbors abundant genetic resources with obvious interspecific and intraspecific variation in Psa susceptibility. For commercial cultivars, yellow-fleshed *A. chinensis* cv. ‘Hort16A’ and cv. ‘Hongyang’ are highly susceptible to Psa, whereas *A. deliciosa* cv. ‘Hayward’ exhibits moderate to high resistance. Wild *Actinidia* species generally carry superior Psa resistance [15,16,17]. Previous studies have identified wild germplasms with high resistance, including *A. arguta*, *A. eriantha*, *A. kolomikta*, *A. callosa* var. *discolor*, and *A. melanandra*, which can act as potential resistance donors in kiwifruit breeding [18,19,20,21]. Ploidy level is positively correlated with Psa resistance: hexaploid and octoploid kiwifruit accessions show significantly higher resistance than diploid and tetraploid ones. Phenotypically resistant kiwifruit germplasms usually showed higher activities of peroxidase (POD) and phenylalanine ammonia-lyase (PAL) [22], activated sucrose metabolism [17], and optimized stomatal density [23] after Psa inoculation, which jointly activate host immunity to block Psa colonization and invasion.

Current integrated management strategies for Psa mainly include quarantine, agronomic regulation, chemical control, and biological control. In commercial production, accurate molecular detection and standardized quarantine screening of propagation seedlings are essential [24], which prevents the introduction of pathogen-carrying seedlings into disease-free orchards and reduces primary infection risks [3,25]. Agronomic control optimizes orchard microclimate via canopy pruning to reduce canopy density, rational tree vigor management [20], balanced fertilization with reduced nitrogen input [26], and improved ventilation and humidity control to disrupt conditions that favor Psa infection. Combined region-specific cultivation practices and ecological early-warning systems can further improve field control efficacy [24,27].

Conventional chemical control relies heavily on copper bactericides and synthetic antibiotics. Agricultural antibiotics are banned in most EU countries, and their application is only permitted on non-bearing young kiwifruit plants in a small number of authorized regions [3,28]. Tetracyclines merely relieve disease symptoms transiently rather than eliminating endophytic Psa colonizing vascular tissues to achieve long-term vine recovery [8,29]. Even with rapid in planta suppression of Psa, these chemical agents still bring two critical drawbacks: accelerated pathogen resistance selection and agrochemical residue accumulation in the environment [8,30]. Environment-friendly biocontrol has emerged as a sustainable alternative, consisting of three main categories: plant defense elicitors, antagonistic microorganisms, and natural bioactive compounds. Commercial elicitors such as acibenzolar-S-methyl (ASM) [31,32] and chitosan [33] trigger systemic acquired resistance (SAR) in kiwifruit. Antagonistic microbes (*Bacillus* spp., *Pseudomonas* spp.) and lytic bacteriophages inhibit Psa proliferation through niche competition and bacterial lysis [28,34,35,36,37]. In addition, plant essential oils [38] and antimicrobial peptides (AMPs) [39,40] display strong antibacterial activity against Psa, with verified efficacy in laboratory and field trials [24,41].

Nevertheless, conventional field management has inherent limitations that hinder long-term sustainable Psa suppression. Repeated copper application leads to heavy metal accumulation in soil and adaptive resistance in Psa, while excessive antibiotic use drives the emergence of highly resistant virulent strains that can decrease control efficiency and endanger food safety [30,42]. Biocontrol agents often show unstable field performance under fluctuating environmental conditions, and innovative biocontrol products, such as bacteriophages, AMPs, and plant extracts, have not been widely commercialized [24,40]. Meanwhile, existing pathogen forecasting and precise detection models are vulnerable to climate change and combined biotic–abiotic stresses, reducing early warning accuracy [43]. Importantly, Psa populations continuously evolve novel virulent pathotypes and pesticide-resistant strains to overcome existing management measures [20]. Accordingly, breeding resistant kiwifruit cultivars is the most cost-effective, eco-friendly, and sustainable long-term strategy for Psa management.

To develop Psa-resistant kiwifruit cultivars, obtaining elite sources of resistance is essential. This study adopted a detached shoot inoculation system to evaluate Psa resistance through three consecutive years across 86 *Actinidia* accessions, aiming to screen germplasm with relatively stable disease resistance. The elite resistant germplasm screened in this study and the clarified differential biochemical defense responses could provide key germplasm and a physiological and theoretical basis for subsequent resistance gene mining and disease resistance breeding.

## 2. Results

### 2.1. Quantitative Evaluation of Psa Resistance in 86 Actinidia Accessions Based on Lesion Length

**Figure 1:**
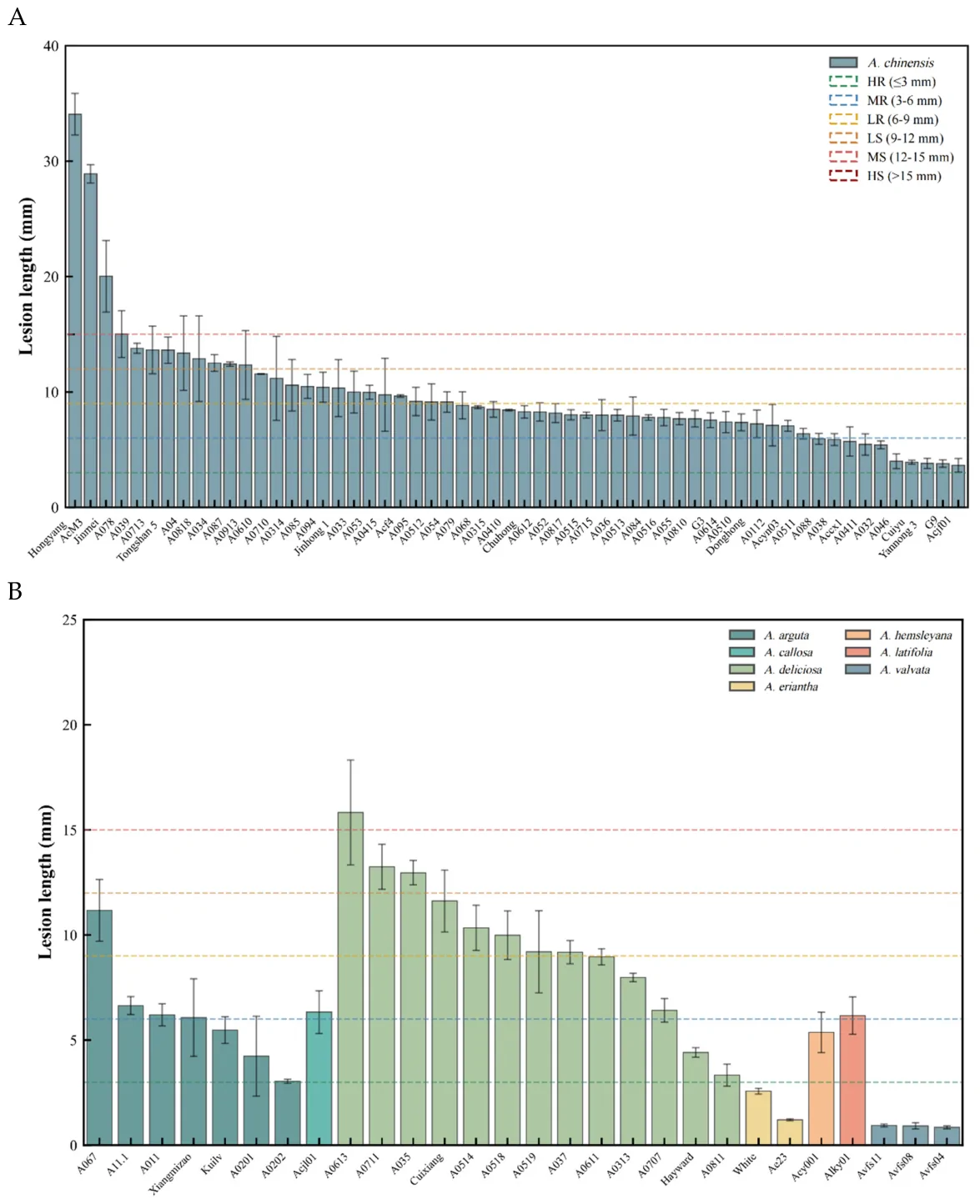
Lesion lengths of different *Actinidia* accessions after inoculation. (**A**) Mean lesion lengths (mm) of 58 *A. chinensis* accessions. (**B**) Mean lesion lengths of 28 accessions from other *Actinidia* species, with different colors representing distinct species. Dashed horizontal lines indicate resistance classification thresholds. Error bars represent standard deviation (SD).

To evaluate resistance levels across *Actinidia* germplasm, lesion length was used as a direct phenotypic indicator of Psa susceptibility. The 58 *A. chinensis* accessions exhibited continuous quantitative variation in disease response. The cultivar ‘Hongyang’ produced lesions exceeding 30 mm and was classified as highly susceptible, whereas ‘Yannong 3’ and Acj01 showed lesion lengths that were ≤3 mm, indicating high resistance (Figure 1A). Most cultivated accessions exhibited moderate to slight susceptibility. Distinct interspecific resistance divergence was observed among the 28 wild *Actinidia* accessions. Accessions from *A. deliciosa* showed generally high susceptibility, and the accession A0613 exceeded the threshold for high susceptibility. In contrast, all *A. valvata* accessions developed lesions that were shorter than 3 mm. Most accessions from *A. arguta* and *A. callosa* were moderately resistant, while accessions from *A. hemsleyana* and *A. latifolia* were moderately susceptible. Additionally, two *A. eriantha* accessions consistently displayed high resistance to Psa, with all individuals falling into the high-resistance category (Figure 1B).

### 2.2. Stem Lesion Phenotypes and Resistance Grading Across 86 Actinidia germplasm

**Figure 2:**
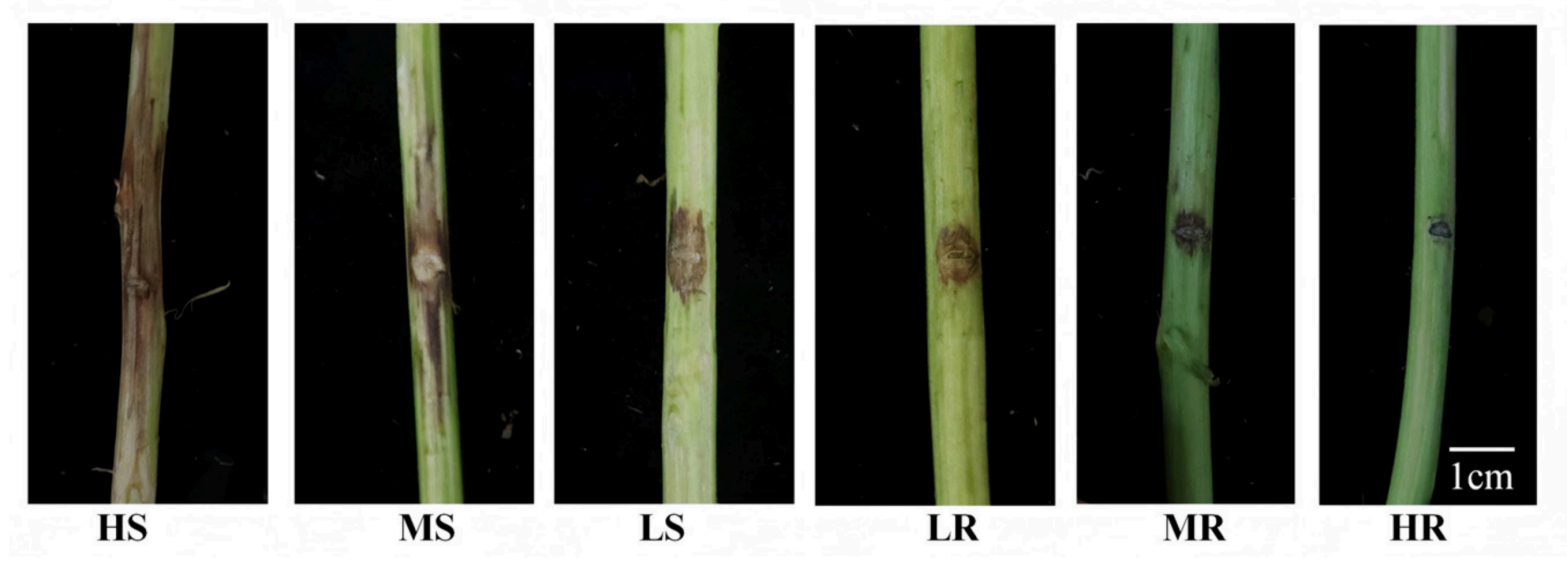
Typical lesion phenotypes of detached shoots from *Actinidia* germplasm with distinct Psa resistance levels at 24 days post inoculation. HR, high resistance; MR, moderate resistance; LR, low resistance; LS, low susceptibility; MS, moderate susceptibility; HS, high susceptibility. Scale bar = 1 cm.

To observe the intuitive disease symptoms of all tested germplasms at the same growth stage, detached shoots of 86 *Actinidia* accessions were inoculated with Psa and photographed at 24 days post inoculation. Obvious differences in lesion expansion were visually observed among different materials. From HR to HS, the necrotic lesion length gradually increased, and the lesion coverage on stems was significantly expanded in highly susceptible accessions such as ‘Hongyang’. All 86 accessions were classified into six continuous resistance levels (HR, MR, LR, LS, MS, HS) according to their average lesion lengths across three years, which was consistent with the phenotypic difference shown in the stem photographs (Figure 2, Table A1).

### 2.3. Categorical Classification for Psa Susceptibility of 86 Kiwifruit Accessions

**Figure 3:**
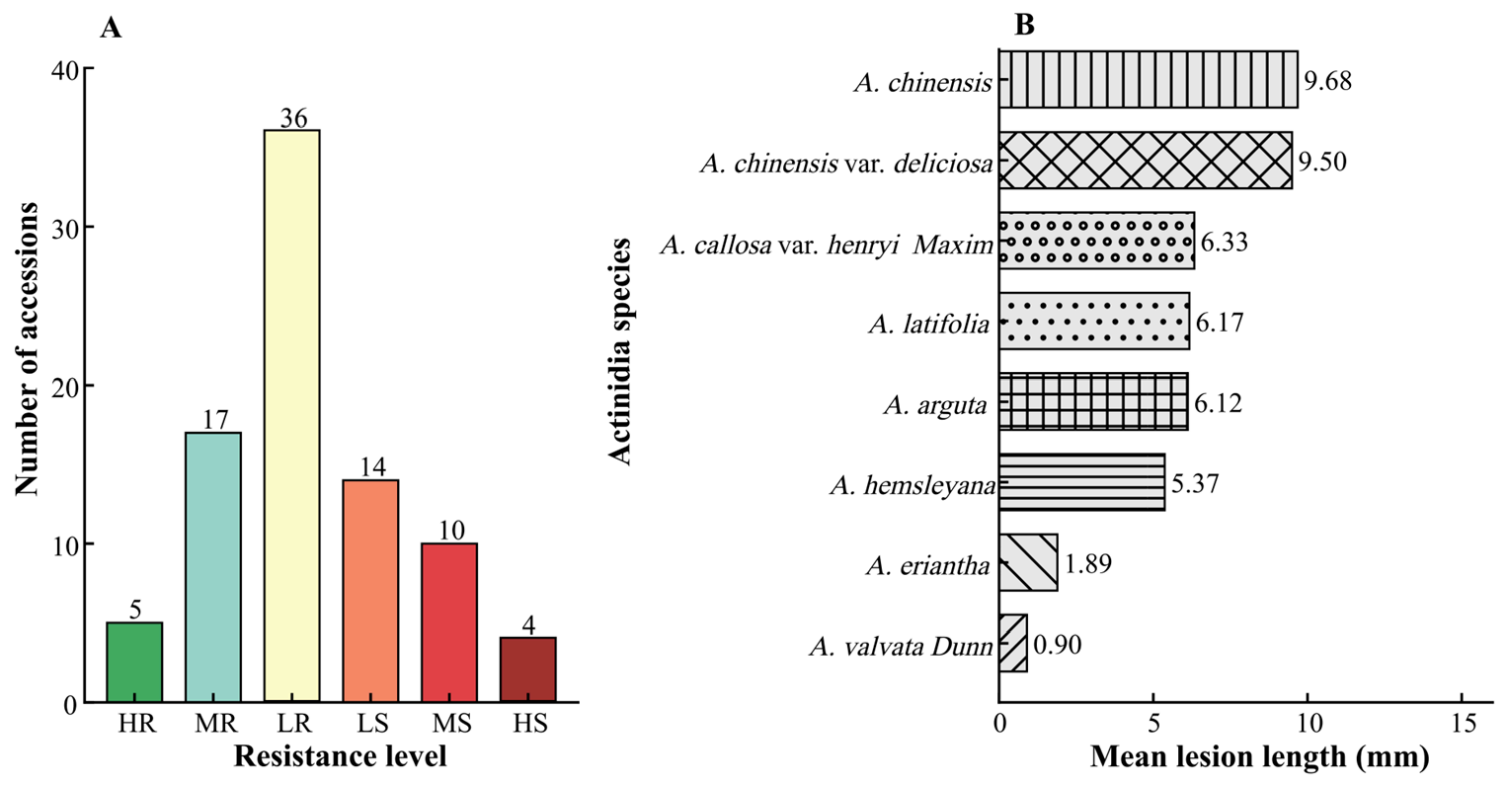
Disease resistance distribution and interspecific lesion length differences among kiwifruit germplasms. (**A**) Quantity distribution of 86 germplasms classified into six resistance grades. (**B**) Comparison of mean lesion lengths among eight *Actinidia* species. HR, high resistance; MR, moderate resistance; LR, low resistance; LS, low susceptibility; MS, moderate susceptibility; HS, high susceptibility. Values are presented as mean lesion length (mm).

Interspecific differences in Psa susceptibility were analyzed based on the average lesion lengths of eight *Actinidia* species and cultivars. Among the 86 tested accessions, five were classified as highly resistant (HR, 5.81%), with lesion lengths ranging from 0.85 to 2.57 mm, mainly including *A. eriantha* and *A. valvata*. Seventeen accessions were moderately resistant (MR, 19.77%), covering multiple species such as *A. chinensis* and *A. arguta*, representing the richest interspecific diversity. Low-resistance (LR) accessions constituted the largest group, with 36 accessions (41.86%). In addition, 14 low susceptibility (LS, 16.28%), 10 moderately susceptible (MS, 11.63%), and four highly susceptible (HS, 4.65%) accessions were identified. All HS accessions belonged to *A. chinensis*, among which ‘Hongyang’ had the maximum mean lesion length of 34.06 mm and was the most susceptible accession (Figure 3A).

Significant interspecific differences in Psa resistance were detected, with the resistance ranking as follows: *A. valvata* > *A. eriantha* > *A. arguta* > *A. deliciosa* > *A. chinensis*. The average lesion lengths of *A. chinensis* and its variety *A. chinensis* var. *deliciosa* were 9.68 mm and 9.50 mm, respectively, indicating moderate to high susceptibility. Nevertheless, several resistant accessions, including ‘Yannong 3’, G9, and A046, were identified within *A. chinensis*, showing great potential for resistance breeding. *A. valvata* and *A. eriantha* exhibited the highest resistance, with average lesion lengths of 0.90 mm and 1.89 mm, respectively, which were significantly lower than those from other *Actinidia* species (Figure 3B).

### 2.4. Stability of Psa Susceptibility Across Years Among Examined Accessions

**Figure 4:**
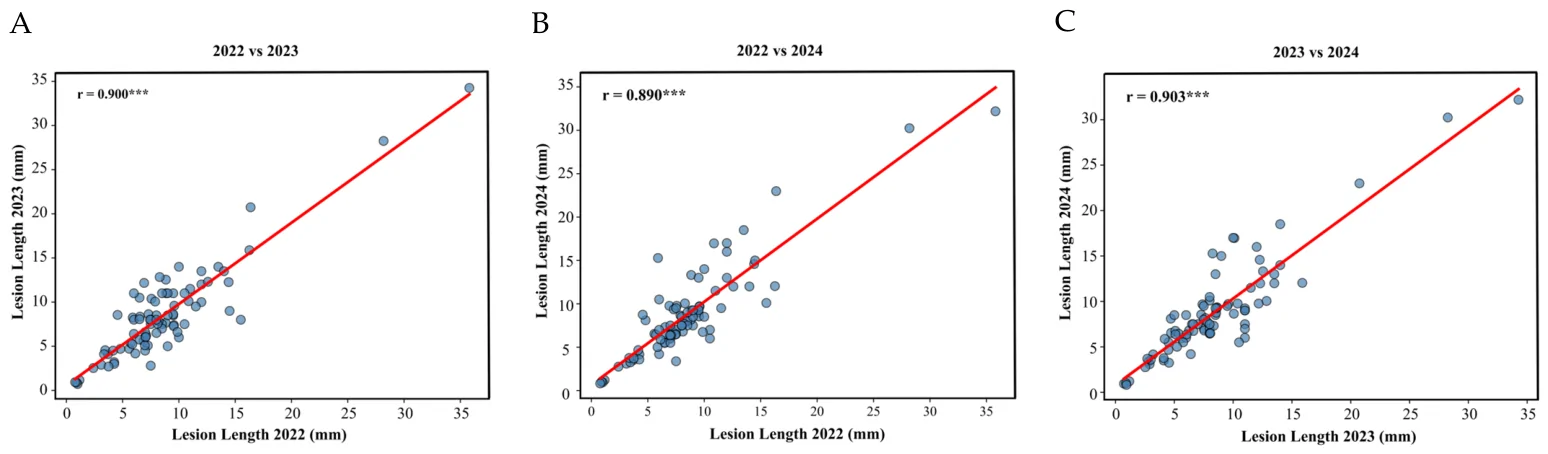
Interannual correlation and stability analysis of disease resistance in *Actinidia* germplasm. (**A**–**C**) Pearson correlation scatter plots of pairwise annual lesion lengths. *** indicates extremely significant correlation at *p* < 0.001.

To evaluate the repeatability of the detached shoot inoculation method and the interannual stability of Psa susceptibility, correlation analyses were performed using lesion length data collected across three consecutive years. Highly significant positive correlations were detected among data from different years (Figure 4), indicating relatively stable Psa susceptibility of tested germplasms across years.

### 2.5. Distribution Characteristics of Lesion Lengths Across Different Resistance Grades

**Figure 5:**
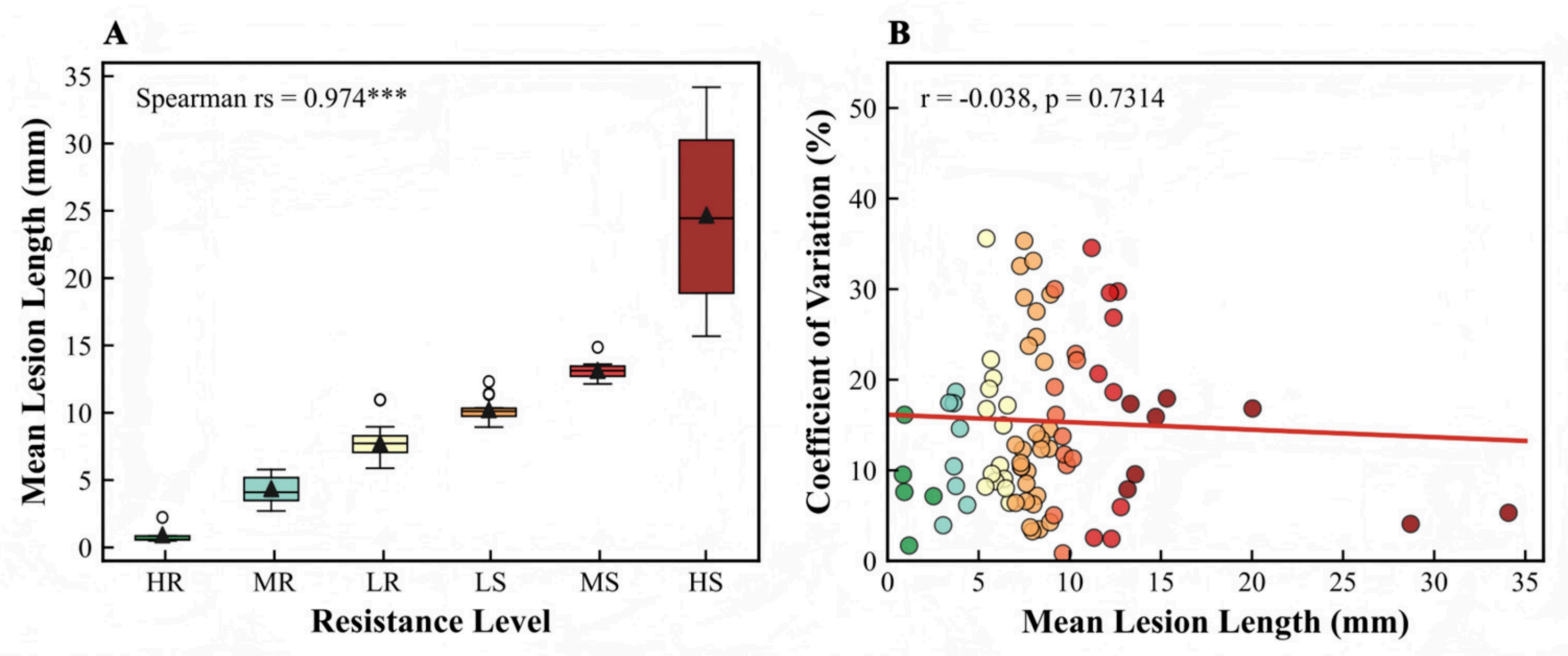
Lesion length distribution and coefficient of variation correlation across different Psa resistance grades. (**A**) Boxplot of mean lesion lengths for six resistance grades with Spearman correlation coefficient. (**B**) Scatter plot showing correlation between average lesion length and coefficient of variation. *** indicates extremely significant difference at *p* < 0.001.

Boxplot analysis was conducted to characterize the lesion length distribution of germplasm within each resistance grade. Spearman rank correlation analysis revealed an extremely significant positive correlation between susceptibility level and mean lesion length (*rs* = 0.974, *p* < 0.001). HR accessions produced the shortest and most concentrated lesions, while HS accessions exhibited substantially longer necrotic lesions (Figure 5A). Pearson correlation analysis between mean lesion length and interannual coefficient of variation (CV) showed no significant linear relationship (*r* = −0.038, *p* = 0.7314) (Figure 5B). CV values fluctuated randomly regardless of lesion length gradient, meaning that strong susceptibility did not correspond to larger interannual phenotypic variation.

### 2.6. Defense-Related Enzyme Activity Changes of Kiwifruit Germplasm with Contrasting Resistance Levels After Psa Inoculation

**Figure 6:**
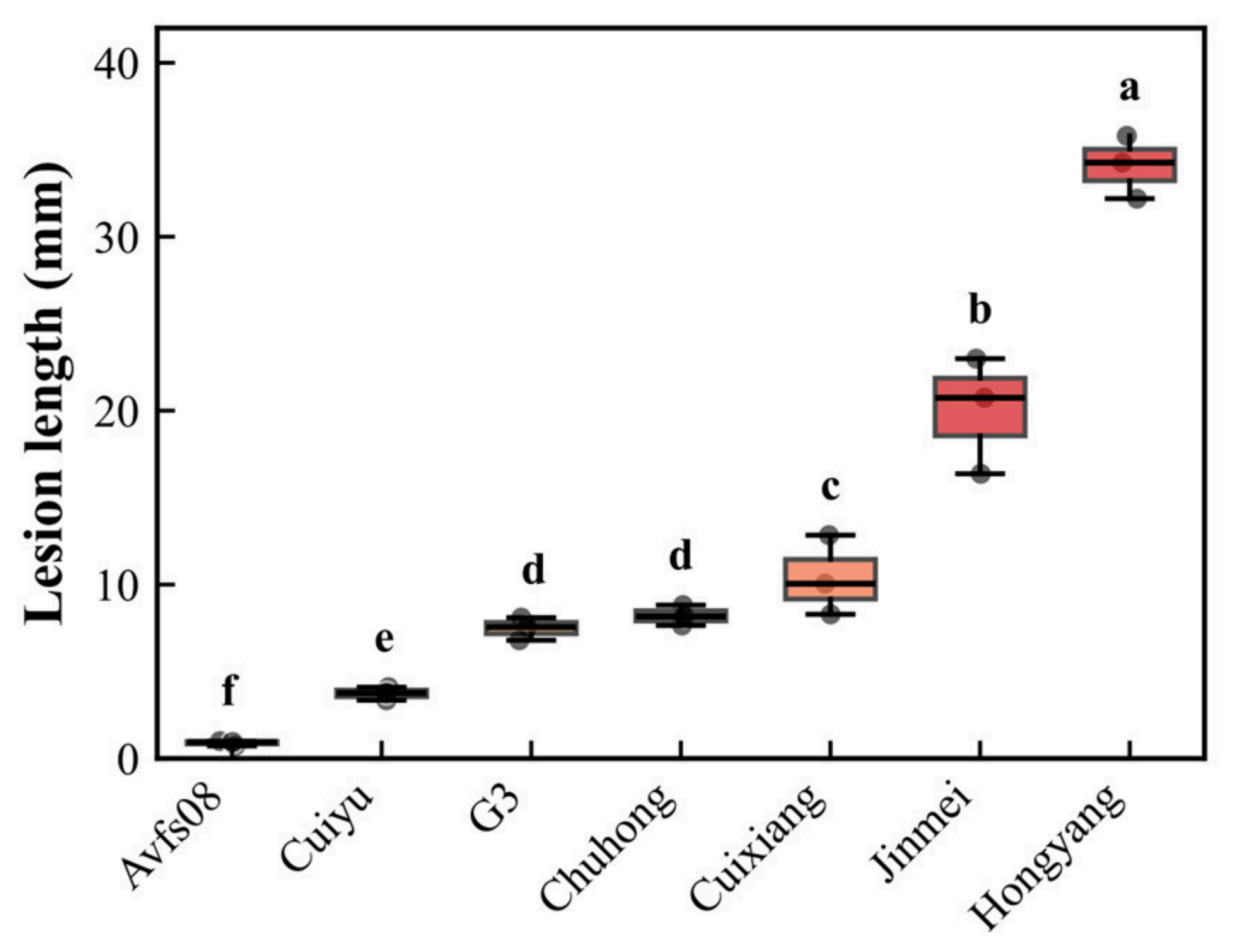
Lesion lengths and resistance classification of seven representative *Actinidia* accessions selected for physiological enzyme analysis. Lowercase letters denote significant differences at *p* < 0.05 (one-way ANOVA, Duncan’s test).

To further verify the rationality of the adopted resistance grading standards and select materials for analyzing physiological responses to Psa infection, seven accessions spanning the full gradient from high resistance to high susceptibility were subjected to defense enzyme activity assays. Their average lesion lengths ranged from 0.92 mm to 34.06 mm, and significant differences in lesion length were detected among the seven tested germplasms (Figure 6).

**Figure 7:**
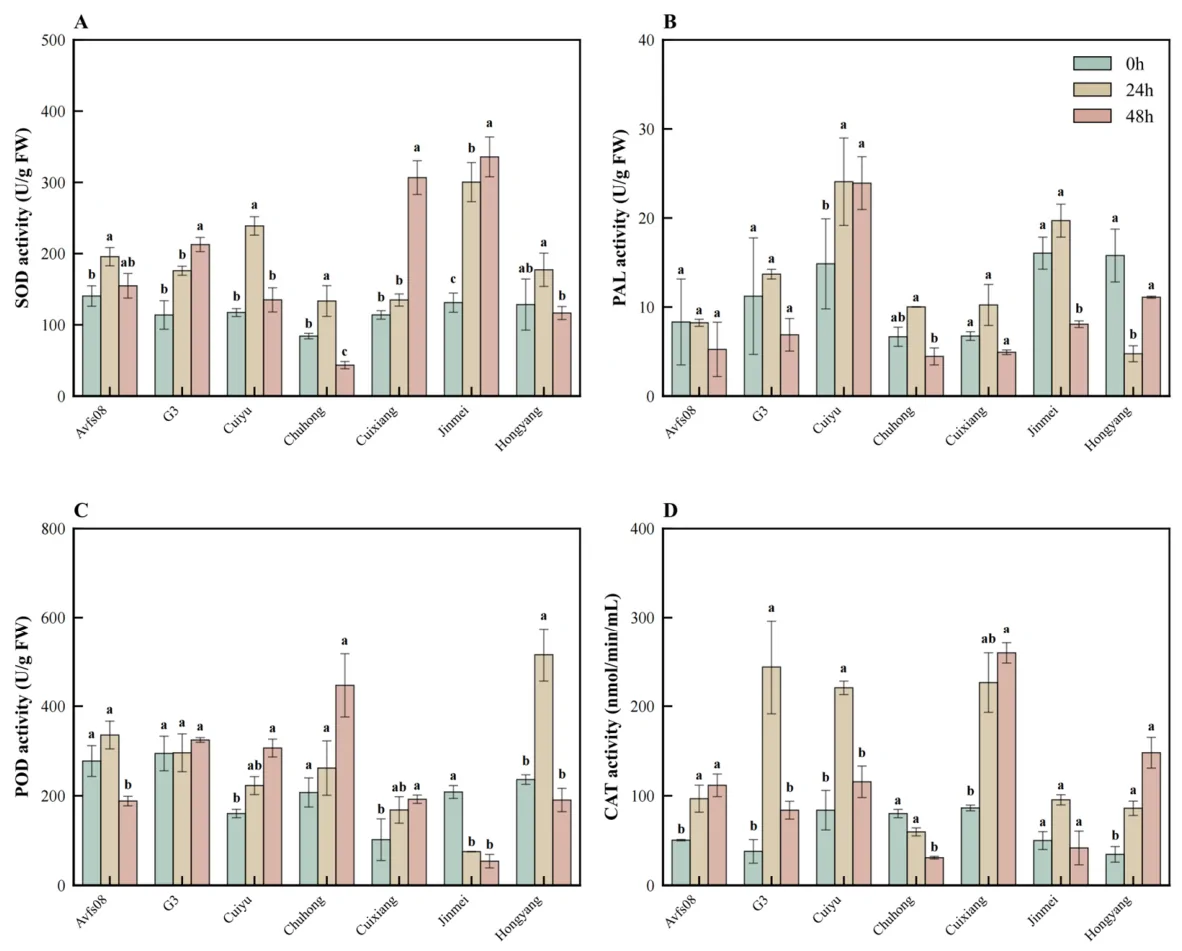
Changes in antioxidant enzyme activities in different *Actinidia* accessions after Psa inoculation. (**A**) SOD activity; (**B**) PAL activity; (**C**) POD activity; (**D**) CAT activity. Data represent mean ± SD (*n* = 3). Different lowercase letters indicate significant differences among time points within each accession (*p* < 0.05).

To explore the distinct physiological responses of kiwifruit germplasm with different resistance levels to Psa infection, the activities of SOD, POD, CAT, and PAL were determined 0, 24, and 48 h post inoculation (hpi). The highly resistant accession Avfs08 showed increased SOD activity at 24 hpi and maintained a stable level thereafter. The moderately resistant cultivar ‘Cuiyu’ exhibited a single-peak SOD trend, whereas the highly susceptible cultivar ‘Hongyang’ showed only a transient SOD elevation. ‘Jinmei’ displayed continuously increasing SOD activity throughout the experimental period. For PAL activity, ‘Cuiyu’ maintained consistently high values at 24 and 48 hpi, while ‘Hongyang’ showed a sharp decline at 24 hpi. For POD activity, ‘Hongyang‘ reached the highest level at 24 hpi, whereas ‘Jinmei‘ exhibited a continuous decrease after inoculation. For CAT activity, most germplasms reached their peak values at 24 hpi, while ‘Cuixiang‘ sustained high CAT activity until 48 hpi (Figure 7).

### 2.7. The Correlation Between Defense-Related Enzyme Activity and Lesion Length

**Figure 8:**
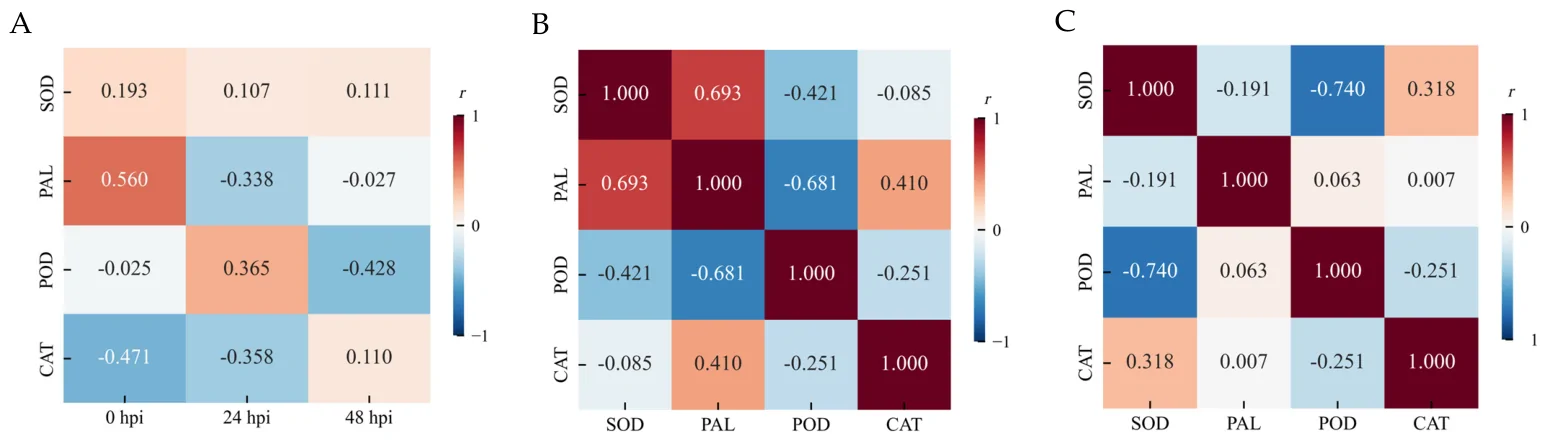
Pearson’s r correlation heat maps. (**A**) Correlation coefficients between four defence-enzyme activities and lesion length at 0, 24, and 48 hpi. (**B**) Inter-enzyme correlation at 24 hpi. (**C**) Inter-enzyme correlation at 48 hpi. Red stands for positive correlation and blue stands for negative correlation. The color-bar label represents the r-coefficient.

Correlation analysis was implemented to quantify the relationship between four defense-related enzyme activities and shoot lesion length, as well as the dynamic inter-enzyme synergistic relationships at 0, 24, and 48 hpi (Figure 8). SOD exhibited weak linear correlations with lesion length at all sampling points, indicating that the independent change of SOD activity could not directly determine the final lesion size. A moderate positive correlation between PAL activity and lesion length was observed at 0 hpi, which revealed that the baseline PAL level was higher in susceptible germplasms. After Psa inoculation, the PAL-lesion length correlation turned negative. The basal CAT activity showed a negative correlation with disease severity at 0 hpi; germplasms with higher initial CAT activity tended to form shorter lesions and carry stronger resistance. POD presented obvious time-dependent correlation shifts, which was positively linked to lesion length at 24 hpi and negatively correlated at 48 hpi.

## 3. Discussion

A stable and reliable resistance evaluation system is essential for germplasm screening and breeding for kiwifruit bacterial canker resistance. Three-year consecutive phenotypic data verified that the stability and reproducibility of the detached shoot inoculation assay were high. The strong interannual correlation further suggests that kiwifruit Psa resistance is mainly genetically controlled, while environmental variation has a relatively minor effect on phenotypic performance. Therefore, the average lesion length across three years can accurately reflect the inherent genetic resistance level of each accession. Significant positive interannual correlations were consistent with previous findings by Wang et al. [16,17]. Wild resistant germplasm maintained stable resistance across years, while Psa susceptibility fluctuation in susceptible cultivars may have stemmed from environmental factors rather than genotypic variation [19,44,45]. Compared with field natural infection assessment, this in vitro system eliminates disturbances from climate, pathogen density, and field management to improve data credibility [46], serving as a robust tool to screen for resistant germplasm.

Global screening for Psa resistance within kiwifruit germplasm varies greatly across research institutions in China, New Zealand, Italy, and other major kiwifruit-producing countries, showing both consistent resistance trends and obvious regional differences in screening objects and evaluation pipelines. Overseas breeding programs predominantly focus on cultivated *A. chinensis* and *A. deliciosa* commercial cultivars and uniformly showed that diploid yellow-fleshed cultivars such as ‘Hort16A’ were highly susceptible, while tetraploid germplasm displayed enhanced field tolerance to Psa; nevertheless, overseas germplasm banks lack systematic large-scale identification of wild species from Sect. Leiocarpae including *A. valvata* and *A. eriantha*, which are the core high-resistance donors discovered in our work [16,21,47]. By contrast, domestic research in China prioritizes native wild *Actinidia* resources. Multiple large-scale germplasm surveys consistently rank *A. valvata*, *A. eriantha*, and some *A. arguta* lines as elite resistant materials [1,48], which is consistent with the interspecific resistance gradient observed among the 86 accessions tested herein.

Distinct interspecific resistance divergence existed within the genus *Actinidia*, which cannot be simply explained by species taxonomy. Accessions from *A. valvata* and *A. eriantha* showed drastically limited lesion expansion, accompanied by rapid callus formation and normal axillary bud sprouting after inoculation, demonstrating integrated physical barrier and biochemical defense responses and qualifying them as potential resistant donors. Most *A. arguta* accessions showed moderate or low resistance, whereas *A. chinensis* and its variants were generally susceptible, which agreed with published reports [16,21,22]. Although wild resistant species carry superior resistance alleles, gradual resistance breakdown in some wild accessions reminds us of the adaptive virulence evolution of Psa strains [49,50]. Obvious intraspecific variation was also observed: moderate resistance was identified in *A. arguta* accessions A0201 and A0202, while A067 showed low resistance. Within the usually susceptible species *A. chinensis*, commercial cultivar ‘Hongyang‘ was highly susceptible, but ‘Cuiyu’, ‘Yannong 3’, and G9 showed moderate resistance. These elite resistant accessions can be utilized to avoid linkage drag and asynchronous maturation commonly occurring in distant interspecific crossing. Highly resistant accessions, including *A. eriantha* Ae23 and *A. valvata* Avfs04, can be used as potential breeding parents. Notably, *A. valvata* was screened as a highly resistant species against Psa in the present study. Previous studies have reported that certain accessions of *A. valvata* exhibit strong tolerance to kiwifruit vine decline syndrome (KVDS), a complex stress triggered by waterlogging and soil-borne oomycetes [1,2,51]. Such dual resistance may be largely attributed to conserved phenylpropanoid-lignin and broad-spectrum immune pathways, highlighting wild *Actinidia* species as precious resources for breeding cultivars resistant to multiple biotic and abiotic pressures.

Pearson’s correlation analysis further revealed distinct time-dependent associations between four defense-related enzyme activities and lesion-length phenotype, as well as dynamic synergistic relationships among antioxidant enzymes at different infection stages. The highly resistant accession Avfs08 induced steady coordinated upregulation of SOD and PAL at 24 hpi, activating the phenylpropanoid pathway via moderate ROS signaling to balance defense response and oxidative damage. In contrast, susceptible ‘Hongyang’ showed an abrupt POD burst coupled with sharp PAL depression; this isolated antioxidant response may fail to trigger lignin synthesis and represented invalid stress feedback. Studies suggest that temporal synergy of defense enzymes matters more than the peak value of individual enzymes for their final resistance [22,52,53]. PAL and POD were significantly positively correlated: PAL synthesizes lignin precursors while POD participates in lignin polymerization and H_2_O_2_ scavenging, and their synergistic action strengthens cell wall physical defense and maintains ROS homeostasis [54,55]. Given the defects of long-term chemical and biological control, exploring native resistant germplasm and clarifying their physiological pathways to fight against Psa infection can be a sustainable strategy for eco-friendly canker management. The resistant accessions identified in this study can provide donors for breeding Psa-resistant kiwifruit cultivars. Further research will focus on key genes in the PAL–POD synergistic cascade to facilitate the elucidation of the resistance mechanism against Psa infection in specific kiwifruit accessions.

## 4. Materials and Methods

### 4.1. Plant Materials

#### 4.1.1. Materials for In Vitro Shoot Resistance Assay

A total of 86 *Actinidia* accessions with diverse geographic origins and genetic backgrounds were used in this study (Table 1). All plants were grown under unified irrigation, fertilization, pruning, and pest management regimes at the Kiwifruit Germplasm Repository of Hunan Academy of Agricultural Sciences and the Chang’an Teaching and Research Base of Hunan Agricultural University. One-year-old fully lignified dormant shoots were collected annually from December to January across three consecutive dormant seasons (2022–2024) for detached shoot assay.

**Table 1:** Information on 86 *Actinidia* accessions used in this study.

Species	Number
*A*. *chinensis*	58
*A. chinensis* var. *deliciosa*	13
*A*. *arguta*	7
*A*. *valvata*	3
*A*. *eriantha*	2
*A*. *callosa* var. *henryi*	1
*A*. *hemsleyana*	1
*A*. *latifolia*	1
Total	86

#### 4.1.2. Materials for in Planta Spray Inoculation

Seven accessions representing a continuous resistance grade (from highly resistant to highly susceptible) were selected from the 86 accessions based on a detached shoot assay. Uniform-sized *A. valvata* (Avfs08) plants propagated by cuttings were adopted as the rootstock material. This accession Avfs08 was identified from wild accessions with molecular-marker identification and disease-resistance assessment. All Avfs08 plants were produced via cutting propagation and received no grafting operation. The seven selected scion materials excluded Avfs08. Scions from these seven accessions were grafted onto uniform Avfs08 rootstocks, potted, and grown with standardized substrate and management in a greenhouse at Hunan Agricultural University prior to whole-plant inoculation.

#### 4.1.3. Pathogen Strains and Pathogenicity Validation

*Pseudomonas syringae* pv. *actinidiae* (Psa) strain Psa-FH8 was isolated from naturally infected ‘Hongyang’ kiwifruit in Fenghuang County, Hunan Province, validated via morphological observation, 16S rDNA sequencing, and pathogenicity assays, and preserved at −80 °C. Biovar 3 reference strain C48 was generously provided by Prof. Caihong Zhong, Wuhan Botanical Garden, Chinese Academy of Sciences.

#### 4.1.4. Bacterial Suspension Preparation

Psa-FH8 was streaked on LB agar and incubated at 25 °C for 48~72 h. Single colonies were cultured in liquid LB at 25 °C, 200 rpm, until the mid-logarithmic phase. Bacterial pellets were washed twice with sterile distilled water. An OD_600_–CFU standard curve was constructed. Briefly, serially diluted bacterial suspensions were measured for OD_600_ absorbance, and viable colony counts were obtained via LB-agar plate cultivation to establish the linear calibration relation. This curve was adopted for the spectrophotometric calibration of subsequent inoculum concentration.

Differentiated inoculum-suspension concentrations were adopted for detached shoots and intact plants owing to their divergent tissue structure and physiological state, according to the standard Psa greenhouse inoculation scheme [56]. A higher-concentration bacterial suspension (1 × 10^9^ CFU/mL) was applied to lignified excised shoots to induce stable canker lesions, whereas a 1 × 10^8^ CFU/mL suspension was prepared for whole-plant spray inoculation. All bacterial suspensions were freshly prepared right before each inoculation operation.

### 4.2. Experimental Methods

#### 4.2.1. Resistance Grading Criteria for Detached Shoots

Resistance classification of the 86 accessions was optimized based on 24 dpi lesion length distribution and field disease evaluation, referring to Zhu et al. [57]. Six resistance categories were defined to precisely resolve inter-genotypic resistance divergence (Table 2).

#### 4.2.2. Detached Shoot Inoculation and Lesion Measurement

The inoculation protocol was modified from Wang et al. [16]. Uniform 1-year-old shoots (base diameter 0.8–1.0 cm, length 30 cm) were surface-sterilized with 70% ethanol, rinsed, air-dried, and sealed with paraffin at cut ends. Ten biological replicate shoots were prepared for each *Actinidia* accession. A wound penetrating the xylem was made at the midpoint of each shoot, followed by immediate application of 10 μL Psa suspension (1 × 10^9^ CFU/mL). Shoots were incubated at 16 °C under a 12 h light/12 h dark cycle with high humidity; sterile water served as the negative control. At 24 days post inoculation (dpi), maximum lesion length was measured with a digital caliper (0.01 mm precision), and the mean value of the three most severely diseased shoots per accession was adopted for resistance evaluation.

#### 4.2.3. Whole-Plant Spray Inoculation and Time-Course Sampling

For in planta defense response analysis, adjusted Psa suspension (1 × 10^8^ CFU/mL) supplemented with 0.05% Silwet-L77 was sprayed evenly on the abaxial leaf surface of uniform potted seedlings. The 1 × 10^8^ CFU/mL inoculum concentration complied with the standardized spray-inoculation method for potted kiwifruit seedlings [56]. Plants sprayed with surfactant-containing sterile water were isolated as blank controls to avoid cross-contamination. Greenhouse conditions were set to ≥85% relative humidity, 16–20 °C, 12 h light/dark. Leaf tissues were sampled at 0, 24, 48, and 72 h post inoculation (hpi), pooled from three individual plants per accession with three biological replicates, and stored at −80 °C for later physiological assays.

#### 4.2.4. Antioxidant and Defense Enzyme Activity Assays

The activities of superoxide dismutase (SOD), peroxidase (POD), catalase (CAT), and phenylalanine ammonia-lyase (PAL) were quantified using commercial assay kits (Abbkine Scientific Co., Ltd., Wuhan, Hubei, China) strictly following the manufacturer’s protocols. A total of 0.1 g of fresh-leaf tissue was weighed for each enzyme-activity analysis. Three biological replicates were used for all samples, and enzyme activity was expressed as U per gram of fresh weight (U/g FW).

## 5. Conclusions

In this study, 86 *Actinidia* accessions were evaluated for *Pseudomonas syringae* pv. *actinidiae* (Psa) resistance through three consecutive years using a detached shoot inoculation assay. Obvious interspecific resistance differentiation was confirmed: accessions from *A. valvata* and *A. eriantha* represented stable, highly resistant donors, while the generally susceptible *A. chinensis* still contained usable moderately resistant accessions, including ‘Cuiyu’ and ‘Yannong 3’, enabling intraspecific resistance improvement to circumvent adverse linkage drag from distant interspecific crossing. Temporal coordination of antioxidant and defense enzymes was proven to be the decisive physiological basis for Psa resistance. Resistant accessions synchronously activated SOD and PAL to balance ROS homeostasis and cell wall lignification, whereas susceptible cultivars only produced disordered, ineffective enzymatic bursts. This identified elite resistant kiwifruit germplasm and clarified the PAL–POD synergistic defensive mechanism, providing fundamental breeding materials and theoretical support for the genetic dissection of Psa resistance in kiwifruit and future kiwifruit disease resistance breeding.

## Figures and Tables

**Table 2:** Classification criteria of resistance levels to Psa in *Actinidia* germplasm.

Resistance Level	Lesion Length Range
High Resistance (HR)	lesion length ≤ 3.0 mm
Moderate Resistance (MR)	3.0 mm < lesion length ≤ 6.0 mm
Low Resistance (LR)	6.0 mm < lesion length ≤ 9.0 mm
Low Susceptibility (LS)	9.0 mm < lesion length ≤ 12.0 mm
Moderate Susceptibility (MS)	12.0 mm < lesion length ≤ 15.0 mm
High Susceptibility (HS)	lesion length > 15.0 mm

## Data Availability

The original contributions presented in this study are included in the article. Further inquiries can be directed to the corresponding authors.

## Appendix A

The appendix contains detailed phenotypic and statistical data supporting the resistance identification of *Actinidia* germplasms. Detailed lesion length measurements and corresponding resistance classification information are provided in the following table.

**Table A1:** Lesion length and resistance level classification of kiwifruit germplasms.

Species	Accession ID	2022 (mm)	2023 (mm)	2024 (mm)	Mean ± SD (mm)	Resistance Level
*A. arguta*	A0202	3.05 ± 0.37	2.94 ± 0.32	3.13 ± 0.56	3.04 ± 0.10	MR
*A. arguta*	A0201	6.39 ± 2.42	2.91 ± 0.17	3.39 ± 0.66	4.23 ± 1.90	MR
*A. arguta*	Xiangmizao	5.28 ± 1.45	4.76 ± 0.33	8.17 ± 0.76	6.07 ± 1.84	MR
*A. arguta*	Kuilv	5.58 ± 1.68	4.81 ± 0.27	6.05 ± 1.22	5.48 ± 0.63	MR
*A. arguta*	A011	6.76 ± 1.37	5.71 ± 0.26	6.13 ± 0.58	6.20 ± 0.53	LR
*A. arguta*	A11.1	7.10 ± 1.21	6.26 ± 0.38	6.56 ± 0.38	6.64 ± 0.43	LR
*A. arguta*	A067	11.75 ± 4.33	12.29 ± 0.81	9.48 ± 1.32	11.17 ± 1.47	LR
*A. callosa* var. *henryi*	Acjl01	7.22 ± 2.60	5.24 ± 0.39	6.54 ± 0.69	6.33 ± 1.01	LR
*A. chinensis*	Acjf01	4.25 ± 1.08	3.08 ± 0.32	3.59 ± 0.52	3.64 ± 0.59	MR
*A. chinensis*	Cuiyu	3.84 ± 0.59	4.12 ± 0.61	3.78 ± 0.40	3.91 ± 0.18	MR
*A. chinensis*	G9	3.75 ± 0.73	4.12 ± 0.46	3.49 ± 0.61	3.79 ± 0.32	MR
*A. chinensis*	A046	4.07 ± 0.78	4.58 ± 0.63	3.31 ± 0.65	3.99 ± 0.64	MR
*A. chinensis*	Yannong 3	4.03 ± 1.32	3.31 ± 0.38	4.09 ± 0.48	3.81 ± 0.43	MR
*A. chinensis*	A032	5.79 ± 0.47	5.31 ± 0.24	5.14 ± 0.47	5.41 ± 0.34	MR
*A. chinensis*	A0411	6.33 ± 1.64	4.51 ± 0.59	5.51 ± 0.96	5.45 ± 0.92	MR
*A. chinensis*	A038	5.79 ± 1.19	5.41 ± 0.36	6.42 ± 0.55	5.87 ± 0.51	MR
*A. chinensis*	Accx1	6.46 ± 1.96	6.42 ± 0.89	4.22 ± 0.93	5.70 ± 1.27	MR
*A. chinensis*	A088	6.45 ± 1.35	5.82 ± 0.32	5.54 ± 0.64	5.94 ± 0.47	MR
*A. chinensis*	A0511	6.89 ± 1.61	6.03 ± 0.89	6.21 ± 1.28	6.38 ± 0.45	LR
*A. chinensis*	A055	7.89 ± 2.21	8.07 ± 0.35	7.07 ± 0.61	7.68 ± 0.53	LR
*A. chinensis*	Acyn03	7.07 ± 1.19	6.59 ± 1.00	7.53 ± 1.04	7.06 ± 0.47	LR
*A. chinensis*	A068	8.72 ± 2.52	8.52 ± 0.53	8.76 ± 0.64	8.67 ± 0.13	LR
*A. chinensis*	A0614	7.75 ± 2.25	8.07 ± 0.44	6.36 ± 0.73	7.39 ± 0.92	LR
*A. chinensis*	A0510	8.07 ± 2.01	6.60 ± 0.24	7.41 ± 0.78	7.36 ± 0.73	LR
*A. chinensis*	A0516	8.33 ± 1.91	8.03 ± 0.47	6.98 ± 1.01	7.78 ± 0.71	LR
*A. chinensis*	A0817	8.17 ± 2.07	8.36 ± 0.38	7.52 ± 1.08	8.02 ± 0.44	LR
*A. chinensis*	A0112	7.85 ± 4.13	5.03 ± 1.31	8.45 ± 1.18	7.11 ± 1.80	LR
*A. chinensis*	A0512	9.36 ± 4.30	10.55 ± 0.61	7.47 ± 0.77	9.13 ± 1.56	LR
*A. chinensis*	A084	7.82 ± 1.39	8.01 ± 1.10	7.55 ± 1.30	7.79 ± 0.23	LR
*A. chinensis*	G3	8.15 ± 1.99	7.65 ± 0.21	6.89 ± 0.62	7.56 ± 0.64	LR
*A. chinensis*	A0810	8.50 ± 2.20	7.14 ± 0.29	7.41 ± 0.86	7.68 ± 0.72	LR
*A. chinensis*	Donghong	8.61 ± 4.00	6.68 ± 0.38	6.43 ± 0.90	7.24 ± 1.20	LR
*A. chinensis*	A0515	8.27 ± 1.24	7.97 ± 0.39	7.75 ± 0.70	8.00 ± 0.26	LR
*A. chinensis*	A036	8.52 ± 1.60	7.54 ± 1.12	7.89 ± 1.01	7.98 ± 0.50	LR
*A. chinensis*	A0513	9.15 ± 4.14	6.01 ± 1.34	8.56 ± 1.33	7.91 ± 1.66	LR
*A. chinensis*	A052	7.88 ± 2.12	7.52 ± 0.55	9.09 ± 0.99	8.16 ± 0.82	LR
*A. chinensis*	A095	9.07 ± 3.09	8.01 ± 1.38	10.47 ± 1.61	9.18 ± 1.23	LR
*A. chinensis*	Chuhong	8.79 ± 1.71	7.71 ± 0.32	8.33 ± 1.19	8.28 ± 0.54	LR
*A. chinensis*	A0410	8.36 ± 1.63	8.52 ± 1.36	8.40 ± 1.59	8.43 ± 0.08	LR
*A. chinensis*	A0315	7.74 ± 2.42	8.70 ± 0.35	9.04 ± 1.01	8.49 ± 0.67	LR
*A. chinensis*	A0612	9.01 ± 2.92	7.42 ± 0.39	8.37 ± 1.04	8.27 ± 0.80	LR
*A. chinensis*	A0715	9.52 ± 3.35	7.53 ± 1.47	6.93 ± 1.31	7.99 ± 1.35	LR
*A. chinensis*	A0710	9.92 ± 2.80	8.39 ± 0.39	15.23 ± 1.18	11.18 ± 3.65	LR
*A. chinensis*	A054	8.81 ± 3.16	10.15 ± 0.49	8.44 ± 1.32	9.13 ± 0.89	LR
*A. chinensis*	A079	9.48 ± 3.00	7.47 ± 0.27	9.58 ± 1.07	8.84 ± 1.17	LR
*A. chinensis*	A033	11.06 ± 3.99	11.02 ± 2.48	7.88 ± 0.90	9.99 ± 1.82	LS
*A. chinensis*	A0415	12.03 ± 4.25	11.04 ± 2.46	6.18 ± 1.63	9.75 ± 3.16	LS
*A. chinensis*	A053	10.25 ± 3.25	10.38 ± 0.83	9.27 ± 1.20	9.97 ± 0.61	LS
*A. chinensis*	Acf4	9.74 ± 1.16	9.67 ± 0.43	9.53 ± 0.68	9.65 ± 0.11	LS
*A. chinensis*	A085	11.07 ± 3.85	11.06 ± 2.28	9.27 ± 1.36	10.47 ± 1.04	LS
*A. chinensis*	A094	11.26 ± 3.43	11.04 ± 2.04	8.90 ± 1.51	10.40 ± 1.30	LS
*A. chinensis*	A0314	10.23 ± 4.21	8.53 ± 1.66	12.98 ± 1.87	10.58 ± 2.24	LS
*A. chinensis*	Jinhong 1	12.94 ± 6.96	8.00 ± 1.99	10.08 ± 2.20	10.34 ± 2.47	LS
*A. chinensis*	A0610	11.53 ± 1.34	11.54 ± 1.07	11.61 ± 1.54	11.56 ± 0.04	LS
*A. chinensis*	A034	11.75 ± 4.19	12.63 ± 0.87	13.14 ± 1.61	12.51 ± 0.71	LS
*A. chinensis*	A0818	11.57 ± 4.23	10.04 ± 2.12	17.02 ± 2.59	12.88 ± 3.71	MS
*A. chinensis*	A087	12.60 ± 1.59	12.38 ± 0.39	12.24 ± 1.04	12.41 ± 0.18	MS
*A. chinensis*	A039	13.27 ± 4.42	14.04 ± 2.49	14.02 ± 2.35	13.78 ± 0.44	MS
*A. chinensis*	A0913	12.89 ± 6.57	9.11 ± 2.01	15.00 ± 2.49	12.33 ± 2.98	MS
*A. chinensis*	A04	13.09 ± 4.21	10.27 ± 0.65	16.71 ± 1.42	13.36 ± 3.23	MS
*A. chinensis*	A0713	12.81 ± 2.19	12.12 ± 1.92	16.00 ± 2.43	13.64 ± 2.07	MS
*A. chinensis*	Tongshan 5	14.09 ± 2.53	12.32 ± 0.66	14.47 ± 1.60	13.63 ± 1.14	MS
*A. chinensis*	A078	16.35 ± 2.98	16.04 ± 0.93	12.67 ± 1.49	15.02 ± 2.03	MS
*A. chinensis*	Jinmei	16.61 ± 3.57	20.76 ± 0.96	22.71 ± 1.42	20.03 ± 3.10	HS
*A. chinensis*	AcM3	28.55 ± 2.81	28.32 ± 0.83	29.82 ± 2.19	28.90 ± 0.80	HS
*A. chinensis*	Hongyang	35.75 ± 4.29	34.29 ± 1.32	32.15 ± 2.99	34.06 ± 1.80	HS
*A. chinensis* var. *deliciosa*	A0811	3.72 ± 0.76	2.73 ± 0.35	3.54 ± 0.79	3.33 ± 0.52	MR
*A. chinensis* var. *deliciosa*	Hayward	4.15 ± 0.57	4.60 ± 0.34	4.48 ± 0.47	4.41 ± 0.23	MR
*A. chinensis* var. *deliciosa*	A0707	7.06 ± 1.20	6.08 ± 0.38	6.10 ± 0.70	6.41 ± 0.56	LR
*A. chinensis* var. *deliciosa*	A0313	8.04 ± 1.01	8.15 ± 0.41	7.76 ± 0.75	7.98 ± 0.20	LR
*A. chinensis* var. *deliciosa*	A0519	9.45 ± 4.26	11.03 ± 2.35	7.13 ± 1.91	9.20 ± 1.95	LR
*A. chinensis* var. *deliciosa*	A0611	9.20 ± 1.36	8.51 ± 1.19	9.16 ± 1.30	8.96 ± 0.39	LR
*A. chinensis* var. *deliciosa*	A037	9.76 ± 1.66	8.70 ± 0.35	9.07 ± 0.61	9.18 ± 0.55	LS
*A. chinensis* var. *deliciosa*	A0514	10.86 ± 4.09	11.04 ± 2.13	9.11 ± 1.55	10.34 ± 1.08	LS
*A. chinensis* var. *deliciosa*	A0518	11.29 ± 2.89	9.55 ± 0.60	9.12 ± 1.16	9.99 ± 1.15	LS
*A. chinensis* var. *deliciosa*	Cuixiang	11.91 ± 4.04	12.92 ± 0.77	10.03 ± 1.42	11.62 ± 1.47	LS
*A. chinensis* var. *deliciosa*	A035	12.38 ± 2.63	13.53 ± 2.02	12.98 ± 1.79	12.96 ± 0.58	MS
*A. chinensis* var. *deliciosa*	A0711	14.15 ± 2.91	13.53 ± 2.38	12.06 ± 2.30	13.25 ± 1.07	MS
*A. chinensis* var. *deliciosa*	A0613	14.65 ± 3.16	14.14 ± 0.52	18.71 ± 1.42	15.83 ± 2.49	HS
*A. eriantha*	Ae23	1.17 ± 0.11	1.19 ± 0.12	1.26 ± 0.13	1.21 ± 0.05	HR
*A. eriantha*	White	2.40 ± 0.38	2.64 ± 0.26	2.66 ± 0.40	2.57 ± 0.14	HR
*A. hemsleyana*	Acy001	6.18 ± 1.81	4.31 ± 0.24	5.63 ± 0.49	5.37 ± 0.96	MR
*A. latifolia*	Alky01	6.97 ± 2.47	5.22 ± 0.47	6.33 ± 0.70	6.17 ± 0.89	LR
*A. valvata*	Avfs04	0.78 ± 0.11	0.93 ± 0.08	0.84 ± 0.08	0.85 ± 0.07	HR
*A. valvata*	Avfs11	0.90 ± 0.13	0.89 ± 0.14	1.02 ± 0.12	0.94 ± 0.07	HR
*A. valvata*	Avfs08	1.00 ± 0.41	0.75 ± 0.18	1.02 ± 0.21	0.92 ± 0.15	HR

Note: HR, high resistance; MR, moderate resistance; LR, low resistance; LS, low susceptibility; MS, moderate susceptibility; HS, high susceptibility.
